# Assessing the diversity of zoonotic bacterial agents in rodents and small mammals in Iran

**DOI:** 10.1093/eurpub/ckae132

**Published:** 2025-01-13

**Authors:** Niloofar Rezaie, Mina Latifian, Ahmad Ghasemi, Ahmad Mahmoudi, Neda Baseri, Amir Hossein Omidi, Parisa Esmaeili, Saber Esmaeili, Ehsan Mostafavi

**Affiliations:** Department of Bacteriology, Pasteur Institute of Iran, Tehran, Iran; National Reference Laboratory for Plague, Tularemia and Q Fever, Research Centre for Emerging and Reemerging Infectious Diseases, Pasteur Institute of Iran, Akanlu, Kabudar-Ahang, Hamadan, Iran; National Reference Laboratory for Plague, Tularemia and Q Fever, Research Centre for Emerging and Reemerging Infectious Diseases, Pasteur Institute of Iran, Akanlu, Kabudar-Ahang, Hamadan, Iran; Department of Epidemiology and Biostatistics, Research Centre for Emerging and Reemerging Infectious Diseases, Pasteur Institute of Iran, Tehran, Iran; Department of Biology, Faculty of Science, Urmia University, Urmia, Iran; Department of Biology, Faculty of Science, Urmia University, Tehran, Iran; Department of Bacteriology, Faculty of Medical Sciences, Tarbiat Modares University, Tehran, Iran; National Reference Laboratory for Plague, Tularemia and Q Fever, Research Centre for Emerging and Reemerging Infectious Diseases, Pasteur Institute of Iran, Akanlu, Kabudar-Ahang, Hamadan, Iran; Department of Epidemiology and Biostatistics, Research Centre for Emerging and Reemerging Infectious Diseases, Pasteur Institute of Iran, Tehran, Iran; National Reference Laboratory for Plague, Tularemia and Q Fever, Research Centre for Emerging and Reemerging Infectious Diseases, Pasteur Institute of Iran, Akanlu, Kabudar-Ahang, Hamadan, Iran; Department of Epidemiology and Biostatistics, Research Centre for Emerging and Reemerging Infectious Diseases, Pasteur Institute of Iran, Tehran, Iran; National Reference Laboratory for Plague, Tularemia and Q Fever, Research Centre for Emerging and Reemerging Infectious Diseases, Pasteur Institute of Iran, Akanlu, Kabudar-Ahang, Hamadan, Iran; Department of Epidemiology and Biostatistics, Research Centre for Emerging and Reemerging Infectious Diseases, Pasteur Institute of Iran, Tehran, Iran; National Reference Laboratory for Plague, Tularemia and Q Fever, Research Centre for Emerging and Reemerging Infectious Diseases, Pasteur Institute of Iran, Akanlu, Kabudar-Ahang, Hamadan, Iran; Department of Epidemiology and Biostatistics, Research Centre for Emerging and Reemerging Infectious Diseases, Pasteur Institute of Iran, Tehran, Iran

## Abstract

The purpose of this study was to assess the prevalence of zoonotic bacteria, including *Coxiella burnetii*, *Bartonella* spp., *Rickettsia* spp., *Brucella* spp., *Borrelia* spp., and *Ehrlichia* spp., among small mammalian in Iran. We examined 618 small mammals collected between 2016 and 2020 from different parts of Iran. We extracted DNA from spleen samples and used quantitative real-time PCR to identify specific genes. We found 559 (90.45%) samples infected with at least one of the analyzed pathogens. Among the studied specimens, 86.08% tested positive for *Bartonella* spp., 2.42% for *Ehrlichia* spp., 0.80% for *Borrelia* spp., 0.64% for *C. burnetii*, 0.48% for *Brucella* spp., and 0% for *Rickettsia* spp. *Bartonella krasnovii* (25.81%) and *Bartonella taylorii* (25.81%) were the most prevalent among the Bartonella species. This study identified a rodent infected with *Brucella abortus*. Among the *Borrelia-*positive samples, four out of five were identified as *Borrelia duttonii*. Among the positive cases in the *Ehrlichia* genus, *Ehrlichia canis*, *Candidatus Ehrlichia shimanensis*, and *Neoehrlichia mikurensis* were identified. *Meriones persicus* was the most prevalent captured rodent with 315 specimens (51.22%). Our study revealed that a large proportion of the small mammals analyzed were infected with one or more of the targeted pathogens. *M. persicus* exhibited significant infection rates with *C. burnetii*, *Bartonella* spp., *Ehrlichia* spp., *Brucella* spp., and *Borrelia* spp. This suggests that this rodent species could serve as a crucial reservoir for zoonotic pathogens in Iran.

Key points

- Our research has revealed the presence of various bacterial pathogens infecting rodents in different regions of Iran.
- *C. burnetii*, *Bartonella* spp., *Ehrlichia* spp., *Brucella* spp., and *Borrelia* pathogens showed high prevalence in different regions of Iran.
- *M. persicus*, is introduced as a significant reservoir of mentioned pathogens in this study.

## Introduction

Rodents, the largest group of mammals, are significant reservoirs and carriers of zoonotic diseases. They transmit pathogens to humans through various modes, such as bites, contaminated food and water, inhalation of aerosols, and ectoparasites like fleas, lice, and ticks [1].

Emerging and re-emerging zoonotic diseases represent a major global public health threat. These diseases often exist naturally in animal populations but can mutate or adapt to infect humans, leading to rapid and widespread outbreaks. Monitoring and updating information on their prevalence in animal reservoirs are vital for early detection and warning systems for human health [2]. Many of these diseases are difficult to diagnose and treat. Some of these diseases can remain asymptomatic in animals, making it challenging to detect them before they spread to humans. Moreover, many of these diseases can resist conventional antibiotics and antiviral drugs, making them even more challenging to treat [3]. Therefore, monitoring the prevalence of zoonotic diseases in animal reservoirs is crucial to developing effective prevention and control strategies. By identifying the animal species that are most likely to carry these diseases, public health officials can take steps to reduce the risk of transmission to humans. For example, they can implement vaccination programs for domestic animals or restrict the importation of wild animals known to carry these diseases [4].

*Coxiella burnetii* is a bacterium causing Q fever, a zoonotic infection affecting both animals and humans globally. While domestic herbivores are the main reservoirs, wild animals can also be infected. Human transmission typically happens through inhaling contaminated aerosols. Q fever is prevalent in Iran, with documented cases of acute and chronic infections in humans as well as in domestic animals in recent years [5].

*Bartonella* is spread through vectors like ticks, fleas, and lice, with both wild and domestic mammals acting as hosts. Infected hosts often show mild symptoms or remain asymptomatic. Human infections are primarily caused by *Bartonella bacilliformis*, *Bartonella quintana*, and *Bartonella henselae*, with other species also posing risks [6]. Research on small mammal populations in Iran is lacking.

Rickettsial diseases, caused by various species of *Rickettsia*, are zoonotic illnesses transmitted through arthropod bites, including ticks, lice, mites, and fleas [7]. Currently, 18 species of *Rickettsia* have been known to affect humans. There is evidence of Rickettsial infection in ticks and fleas in Iran, and clinical cases are reported [8].

*Ehrlichia*, a bacterial pathogen, causes illness in humans and a range of domestic and wild animals. Rodents serve as reservoirs for this pathogen. Among *Ehrlichia* species, *Ehrlichia canis* and *Ehrlichia chaffeensis* are more prevalent [9]. Iran is known as an endemic area for *Ehrlichia* infections, with documented cases in livestock [10].

*Brucella*, a gram-negative bacterium, causes brucellosis in animals and humans. Transmission happens through unpasteurized dairy consumption or contact with contaminated animal fluids. In Iran, *Brucella* species like *Brucella melitensis*, *Brucella abortus*, *Brucella suis*, and *Brucella ovis* are important public health concerns [11].

*Borrelia* bacteria primarily reside in rodents and mammals, transmitted to other hosts by arthropod vectors. Lyme disease is spread to humans by hard ticks carrying *Borrelia burgdorferi*, while soft ticks carrying *Borrelia miyamotoi* and *Borrelia recurrentis* cause recurrent fever. Studies have shown rodents to be one of the important carriers of *Borrelia* in Iran [12].

The primary aim of this study is to assess the prevalence of microorganisms, including *C. burnetii*, *Bartonella*, *Rickettsia*, *Brucella*, *Borrelia*, and *Ehrlichia* in small mammals from various parts of Iran through molecular techniques. This research is of particular significance as these microorganisms have the potential to cause illnesses in humans, and yet there is limited data on their frequency in the reservoir population.

## Methods

### Sample collection

**Figure 1.**
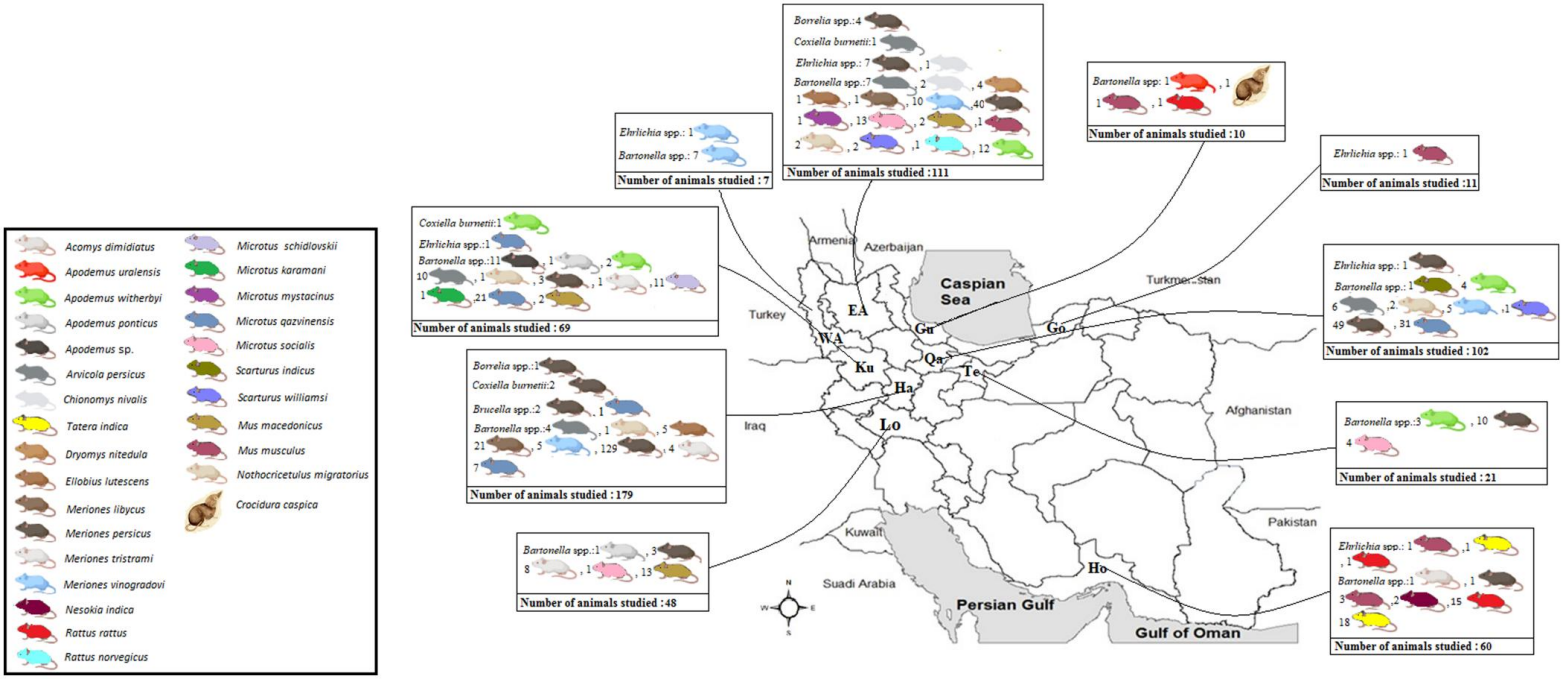
The distribution map showing the number of small mammals studied in each of the targeted 10 provinces and the positive samples of bacteria investigated (*Coxiella burnetii*, *Bartonella*, *Rickettsia*, *Ehrlichia*, *Brucella*, and *Borrelia*) in the samples collected between 2016 and 2020. EA: East Azerbaijan, WA: West Azerbaijan, Ku: Kurdistan, Lo: Lorestan, Ha: Hamadan, Qa: Qazvin, Gu: Guilan, Go: Golestan, Te: Tehran, Ho: Hormozgan.

The spleen samples of small mammals used in this study were sourced from the Biobank of the Research Center for Emerging and Reemerging Infectious Diseases at the Pasteur Institute of Iran. These samples were captured from various regions of Iran between 2016 and 2020 (Fig. 1). Samples were selected based on their relevance to the study’s objectives, focusing on regions with a known high prevalence of plague and tularemia. The sampling methodology and results of previous studies monitoring these diseases have been published in earlier papers and are accessible for reference [13, 14]. Mammals were identified based on their morphological characteristics [15].

The study received official approval from the Research Ethics Committee at the National Institute for the Development of Medical Sciences of the Islamic Republic of Iran. All methodologies and procedures utilized in this study have been granted official authorization. (IR.NIMAD.REC.1399.285).

### DNA extraction from spleen samples

The DNA extraction from the spleen samples used the High Pure PCR Template Preparation kit (Roche Company) according to the instructions provided by the manufacturer. During every stage of DNA extraction, two negative controls were processed alongside spleen samples in each run to monitor for potential contamination.

### Pathogen identification

In this study, the DNA extracted from the spleen of small mammals was analyzed using the TaqMan quantitative real-time PCR (qPCR) technique with specific primers to determine the presence of *C. burnetii*, *Bartonella*, *Rickettsia*, *Brucella*, and *Borrelia*. The SYBR Green qPCR approach was used to identify the 16SrRNA gene of the *Ehrlichia* genus (Supplementary Table S1). In all tests, distilled water was used as a negative control.

### Identification of pathogen species

Specimens yielding positive results in the qPCR examination for each bacterial strain with a quantification cycle (Cq) of less than 25 were selected for species determination. The identification process for each pathogen species proceeded as follows:

To determine the species of *Brucella*, the multiplex PCR method was employed, targeting the genes *BruAb2_0168*, *BMEII0466*, *IS711*, *BR0952*, *BOV_A0504*, *BMEII0635-0636*, and *BMEII0986-0988* [16]. The target genes *rrs*, 16S rRNA, and *gltA* were amplified using the PCR technique to identify the species of *Ehrlichia*, *Bartonella,* and *Borrelia*, respectively (Supplementary Table S2).

Following PCR amplification, the resulting products were subjected to electrophoresis on a 1% agarose gel. Samples displaying distinct bands were then sent to the Genomic Company in Tehran, Iran, for sequencing using the Sanger method. The obtained DNA sequences were compared with the existing sequences in the NCBI database [17] to confirm the type of microorganisms. The sequences were analyzed using Chromas software (chromas version 2.6.6, http://www.technelysium.com.au/chromas.html), and phylogenetic analysis was conducted using mega X 10.1 software (https://www. megasoftware.net).

## Results

The present study collected spleen samples from 615 rodents and 3 insectivores. The most common rodent species were *Meriones* spp. (50.97%), followed by *Microtus* spp. (16.34%), *Apodemus* spp. (7.28%), *Mus* spp. (5.17%), *Arvicola persicus* (4.85%), *Rattus* spp. (4.20%), and *Tatera indica* (3.07%). The specimens were collected from 10 provinces across Iran, with the highest numbers from Hamedan (*n* = 179, 28.96%), followed by East Azerbaijan (*n* = 111, 17.96%), Qazvin (*n* = 102, 16.50%), Kurdistan (*n* = 69, 11.16%), Hormozgan (*n* = 60, 9.70%), Lorestan (*n* = 48, 7.76%), Tehran (*n* = 21, 3.39%), Golestan (*n* = 11, 1.77%), Guilan (*n* = 10, 1.61%), and West Azerbaijan (*n* = 7, 1.13%). Out of the 618 specimens examined, 559 (90.45%) were contaminated with at least one of the examined pathogens, with *Meriones persicus* having the highest infection rate among the captured rodents, with five out of six studied pathogens reported among them.

### Identification of *Bartonella* spp.

Based on the results, 86.08% (*n* = 532) of the examined small mammals tested positive for *Bartonella*. The pathogen exhibited its highest infection rate in Hamedan province, with 176 specimens showing a prevalence of 98.32%, whereas no positive samples were detected in Golestan province (Supplementary Table S4). Among the rodents, *M. persicus* (*n* = 235, 95.52%) had the highest number of positive *Bartonella* cases, with 235 specimens (95.52%) (Supplementary Table S5).

**Figure 2.**
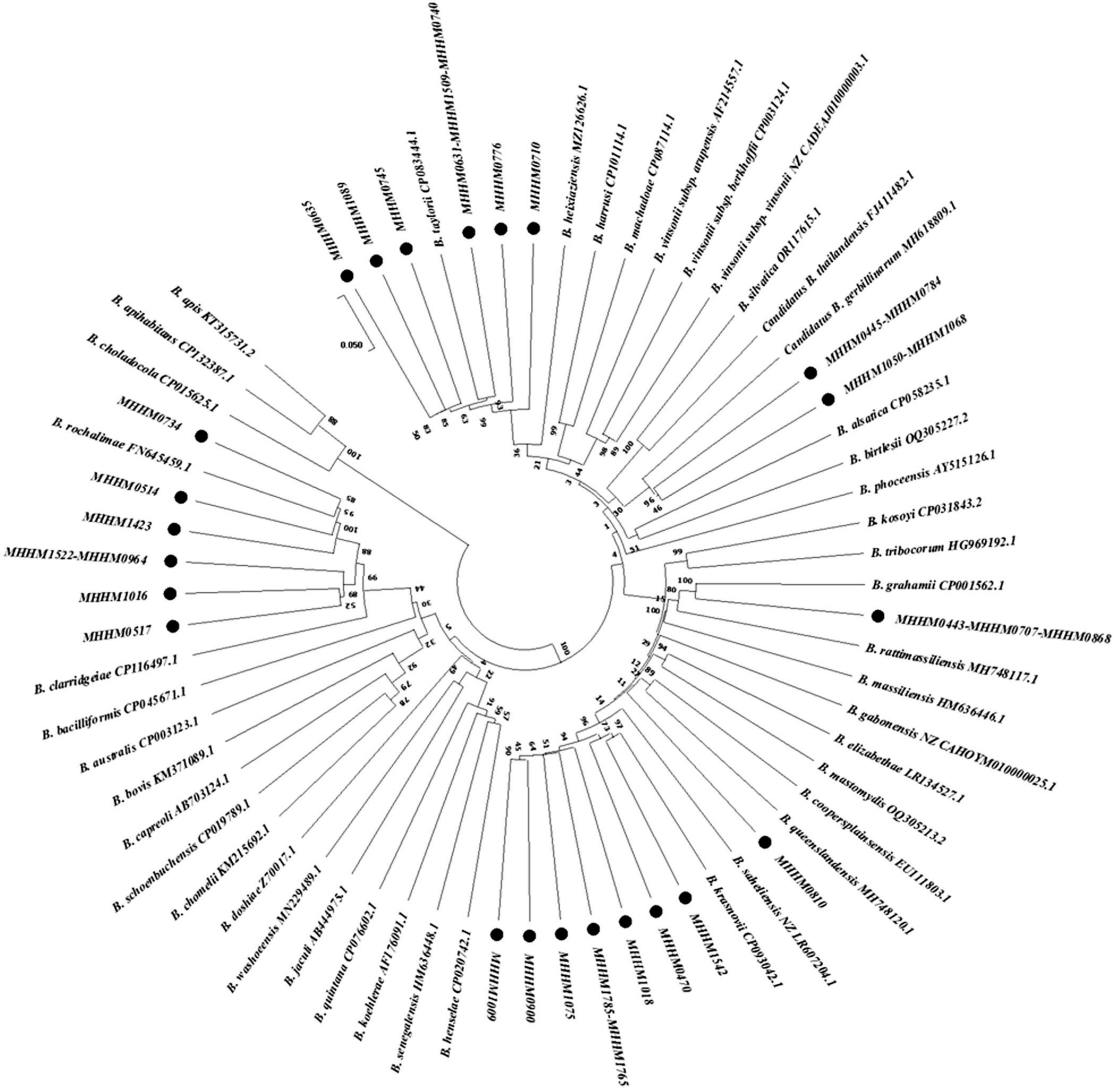
Phylogenetic trees based on the nucleotide sequences of *gltA* gene sequences (790 bp) of *Bartonella* species using the maximum-likelihood (ML) method algorithm (Tamura–Nei model) identified among samples of small mammals in Iran. The test was performed with Bootstrap (1000 repetitions) by MEGA X 10.1 software. Our samples in this study are shown as black circles.

Among the 31 identified specimens from the genus *Bartonella*, *B. krasnovii* (*n* = 8, 25.81%) and *B. taylorii* (*n* = 8, 25.81%) exhibited the highest percentages, followed by *B. rochalimae* (*n* = 7, 22.58%), Candidatus *B. gerbillinarum* (*n* = 4, 12.90%), *B. grahamii* (*n* = 3, 9.68%), and a single sample with *B. queenslandensis* (3.23%) infection (Fig. 2).

### Identification of *Ehrlichia* spp.

Fifteen specimens (2.42%) were found to be infected with the *Ehrlichia* pathogen. The highest number of positive cases was recorded in East Azerbaijan province (*n* = 8, 7.20%). Conversely, no positive cases were observed in Kurdistan, Lorestan, Tehran, and Guilan provinces (Supplementary Table S4). The maximum number of positive cases was also observed in *M. persicus* (*n* = 9, 3.65%) (Supplementary Table S5).

**Figure 3.**
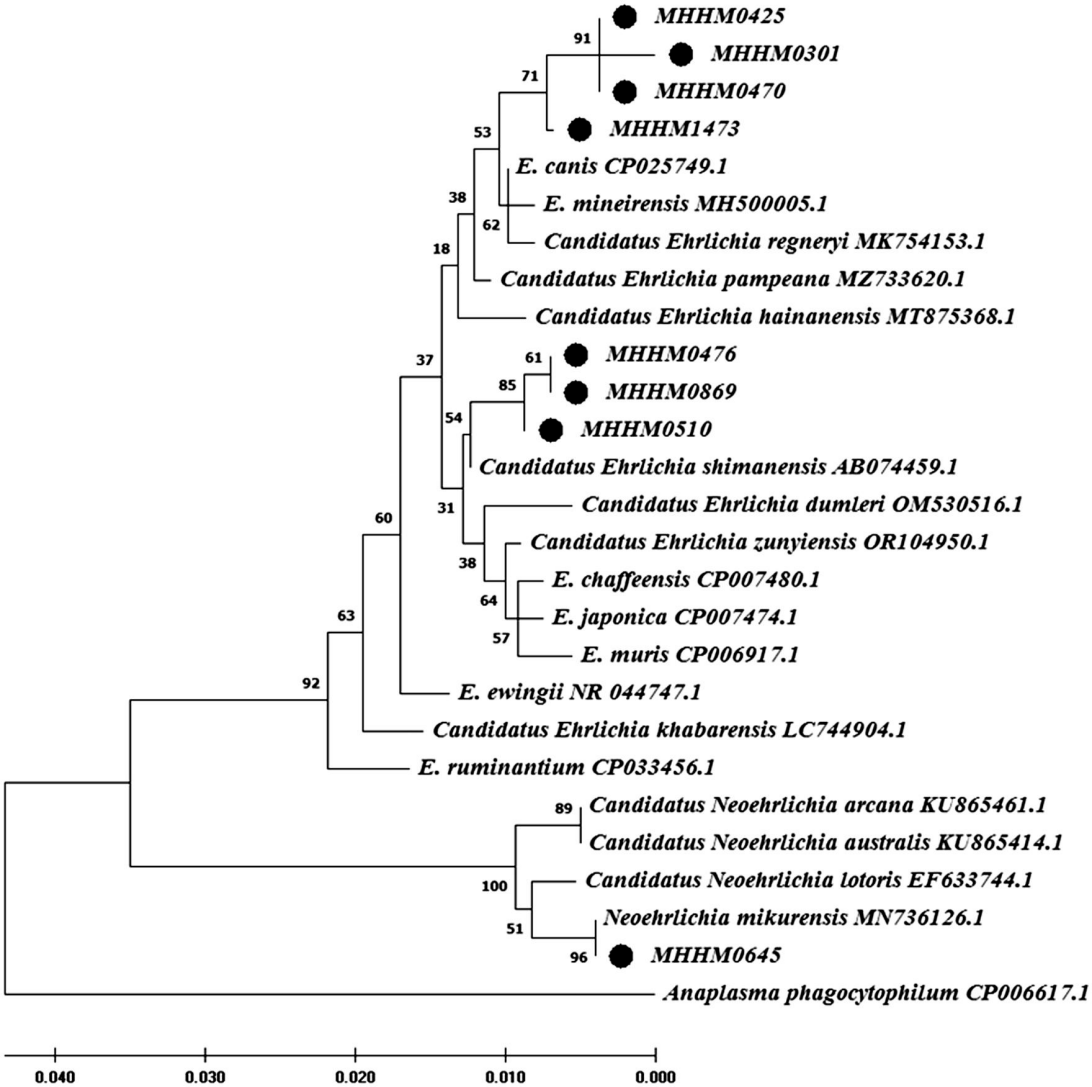
Phylogenetic trees based on the nucleotide sequences of 15SrRNA gene sequences (550 bp) of *Ehrlichia* species using the maximum-likelihood (ML) method algorithm (Tamura–Nei model) identified among samples of mammals in Iran. The test was performed with Bootstrap (1000 repetitions) by MEGA X 10.1 software. Our samples in this study are shown as black circles.

Out of the 15 samples that tested positive for *Ehrlichia*, species identification was successful on eight samples (53.33%), of which four (50%) were identified as *Ehrlichia canis*, three samples (37.5%) were *Candidatus Ehrlichia cf shimanensis*, and one sample (12.5%) was identified as *Neoehrlichia mikurensis* (Fig. 3). In addition, one sample of *M. persicus* (2.38%) from East Azerbaijan showed simultaneous infection with *E. canis* and *B. krasnovii*.

### Identification of *Borrelia* spp.

Four *M. persicus* collected from East Azerbaijan tested positive for *Bartonella duttonii*, and one *M. persicus* collected from Hamadan province tested positive for *B. persica*.

### Identification of *Coxiella burnetiid*

Overall, four samples (0.64%) tested positive for *C. burnetii*. The infected rodents were two *M. persicus* from Hamadan province, one *Apodemus witherbyi* from Kurdistan province, and one *Arvicola persicus* from East Azerbaijan province.

### Identification of *Brucella* spp.

Among the 618 screened spleen samples, three (0.48%) exhibited evidence of the *Brucella* pathogen. Two of these positive samples were from *M. persicus* (0.81%), and one was from *Mi. qazvinensis* (0.58%). Notably, all *Brucella*-infected rodents were from Hamadan province (Supplementary Tables S3 and S5). In addition, one *Microtus qazvinensis* (11.11%) from Hamedan province was found to be co-infected with *Brucella* and *B. rochalimae*.

Among the studied samples, three specimens (0.48%) tested positive for Brucella, all collected from Hamadan province. Two of which were identified as *M. persicus* (0.81%), and one as *M. qazvinensis* (0.58%). Among the three positive samples, one specimen was confirmed to be infected with *Brucella abortus*, while the identities of the remaining positive specimens remained undetermined.

### Identification of *Rickettsia*

None of the studied samples were positive for *Rickettsia*.

## Discussion

In this study, 9 out of 10 small mammals in Iran exhibited at least one of the pathogens studied, and *Bartonella* was the predominant pathogen infecting 86.08% of the specimens.

Rodent-borne diseases can be transmitted to humans through direct and indirect means. In the direct method, infections occur through bites or inhalation of contaminated particles in rodent feces. Indirectly, humans can become infected by consuming food and water contaminated with rodent feces or urine. This makes rodents potential amplifiers for diseases transmitted from rodents to humans by arthropod vectors [1].

Rodents play a crucial role as the primary reservoirs for *Bartonella*, raising concerns about the potential transmission of this pathogen to humans due to their close interaction with these animals. Members of two species of rodent families, Muridae (genera *Apodemus*, *Mus*, *Rattus*, *Bandicota*) and Cricetidae (*Microtus*, *Myodes*= *Clethrionomys*), were very often associated with *Bartonella* spp [18]. Though *B. quintana* has been identified in human cases in Iran [12], *Bartonella* spp. was documented in a cosmopolitan rodent, *Rattus norvegicus*, in Tehran city [19]. Examining the prevalence of this pathogen in wildlife reservoirs attracted little attention. Our investigation revealed that *Bartonella* is the predominant pathogen, infecting a noteworthy 86.08% of the specimens and demanding heightened attention. A study conducted in the United States similarly emphasized the substantial presence of *Bartonella* spp. in 67.5% of diverse rodent species [20]. Moreover, in China, *Bartonella* spp. was detected in 6.4% of the examined rodent population [21].

In our study, we found that ∼50% of the discovered *Bartonella* spp. consisted of *B. taylorii* (25.81%) and *B. rochalimae* (22.58%), both of which have the potential to be transmitted to humans. *Bartonella rochalimae* was identified not only in rodents (*Meriones* spp. and *Microtus* spp.) but also in Eulipotyphla (*Crocidura capsica*), suggesting the putatively wider range of reservoirs involved in its circulation in Iran*. Bartonella taylorii* and *B. queenslandensis*, two of the most frequently detected species worldwide, were found in Iran. *B. taylorii* was detected in *Apodemus flavicollis*, *A. agrarius*, and *Microtus hartingi* from a neighboring country, Turkey [22], and Iran, it is found in a wider range of rodents (*Apodemus* spp., *Microtus qazvinensis*, *Arvicola persicus*, and *Scarturus williamsi*), which occupy different habitats ranging from cultivated fields to riverbanks and dry ecosystems. Moreover, our investigation unveiled the *B. queenslandensis* infection from *Tatera indica* as a new reservoir, for the first time. *B. queenslandensis* was identified first in rodents in Australia [23] but later discovered in 29 small mammal species from more than 13 countries [18]. This study showed again that our current knowledge of *Bartonella* spp. small mammal reservoirs and the distribution of the pathogen are far from final and need comprehensive sampling, especially in neglected regions.

Rodents are the principal reservoirs of *Ehrlichia*-related illnesses in their natural ecosystems. Ehrlichiosis, initially identified as an infectious disease among animals, has now emerged as a zoonotic threat to humans [24]. In our recent investigation, we found *Ehrlichia* spp. in 2.42% of the captured small mammals (15 out of 618 samples), with the majority of positive cases belonging to *M. persicus*. The prevalence of *Ehrlichia* infection in rodents, as reported in this study, is lower than in similar studies conducted in China (17.6%) [25] and Mexico (9.6%) [26]. The findings from this study revealed that a relatively low percentage (0.80%) of the examined small mammals tested positive for *Borrelia* spp. (*Borrelia duttonii and Borrelia persica*). *Meriones persicus* was identified as the sole carrier of *Borrelia*, which is consistent with a recent study conducted in north-eastern Iran in 2017 and 2018 [12]. In broader international research, the prevalence of *Borrelia* infection in rodents and small mammals was reported as 10.7% in America [27], 4.9% in Romania [28], and 1.2% in Thailand [29]. *Coxiella burnetii,* the causative agent of Q fever, is widespread in Iran, with observations across various regions of the country [16]. In this study, 0.64% of the examined small mammals were infected with *C. burnetii*. Interestingly, a separate investigation of *Rattus norvegicus* in Tehran province in 2018 and 2019 revealed a higher prevalence of *C. burnetii* infection in 4% of the rodents [19]. Furthermore, research carried out in Egypt indicated that 6.7% of the analyzed *R. norvegicus* and *R. rattus* were infected with *C. burnetii* [30]. Notably, our study did not detect *C. burnetii* in *Rattus* spp., with most positive cases identified in *M. persicus*. This research is significant as it is the first to pinpoint *M. persicus* as the primary reservoir of Q fever among Iranian wild rodents.

*Brucella* spp. stands out among the microorganisms capable of transmitting from domestic and wild animals to humans. In Iran, rodents that are known to serve as reservoirs for *Brucella* spp. include *R. rattus*, *R. norvegicus*, *M. persicus*, and *Apodemus* spp [31]. In this investigation, the prevalence of *Brucella* spp. among the examined small mammals was 0.48%. *B. abortus* was the predominant infecting species, with the highest number of positive cases identified in *Meriones* species. The incidence of brucellosis infection in this study was notably lower compared to similar investigations conducted both in Iran and globally [11, 32].

In a study carried out in Iran, 39.27% of the rodent samples belonging to *Apodemus* sp. were infected with *Brucella* spp., which belonged to *B. melitensis* (19.93%) and *B. abortus* (9.81%) as the most prevalent species [33]. Similarly, in another analysis conducted in Germany, 14.2% of the rodents were infected with this pathogen [34]. *Rickettsia*, a zoonotic pathogen transmitted through arthropod vectors, is considered an endemic zoonotic pathogen in Iran. However, there is limited knowledge regarding its animal reservoirs. This study is one of the first investigations to determine the presence of this bacterium in rodent and small mammal populations in Iran, and no positive samples for *Rickettsia* were identified. It is worth noting that studies conducted in various regions around the world have reported varying rates of *Rickettsia* infection among rodents, ranging from 0.5% in Slovakia [35] to as high as 17.6% in Poland [36].

In the current study, *M. persicus* exhibited the highest level of contamination among the surveyed rodents. This rodent showcased infection with a diverse range of pathogens, including *C. burnetii*, *Bartonella*, *Ehrlichia*, *Brucella*, and *Borrelia*. Notably, exclusive instances of *Borrelia* infection were identified in *M. persicus*, solidifying its established role as a significant disease reservoir in Iran. Considering *M. persicus*’ recognized status as the primary plague reservoir in Iran [37] and its widespread distribution throughout the country—excluding the southern slope of the Caspian Sea—our research underscores its potential to harbor major zoonotic diseases among rodents in the nation.

In Iran, researchers have identified 79 rodent species and 34 rodent-borne diseases. In eight of these diseases, rodents serve as the primary host, while in others, they act as secondary hosts. Plague, leishmaniasis, and hymenolepis are among the most commonly reported diseases in rodents in Iran. Other diseases, such as salmonellosis, tularemia, borreliosis, bartonellosis, and Crimean-Congo hemorrhagic fever, have been reported to a lesser extent [38].

Understanding the distribution and infection rates of rodents and small mammals, key hosts for emerging zoonotic diseases, is essential for strengthening surveillance systems to detect and respond promptly to human outbreaks. Gathering data on the prevalence and geographic spread of these animals and the diseases they carry enables public health authorities to assess risks to human populations effectively. This information supports targeted surveillance and control measures in high-risk areas, as well as the development of more efficient prevention and intervention strategies. Moreover, comprehending the dynamics of rodent-borne diseases aids in resource allocation and prioritization of intervention areas by public health authorities. Leveraging data from studies on these diseases enhances the monitoring and response capabilities of public health authorities, thereby improving overall responses to zoonotic diseases. This comprehensive approach contributes to more effective disease control and prevention efforts, ultimately mitigating the impact of these diseases on human populations.

Overall, the current study revealed a diverse range of pathogens affecting the rodents under investigation. However, the present study did not explore potential factors like geographical variations, ecological and environmental influences, or host-specific factors that might contribute to the observed differences in prevalence. To gain a more comprehensive understanding of this topic, further research is required.

## Conclusion

Given the significant number of rodents and small mammals found to be carrying *Bartonella* in our research, it is imperative to take action to manage the population of these animals to prevent the transmission of these infectious agents to humans. The presence of *Bartonella* poses a serious risk to human health, making it crucial to conduct thorough investigations to contain the spread of these pathogens and reduce the likelihood of human exposure. Pinpointing the source of these pathogens is essential for promptly and efficiently addressing these concerns.

## Supplementary Material

ckae132_Supplementary_Data

## Data Availability

The data will be shared on reasonable request to the corresponding author.
