# Supplementary material for: Assessing the diversity of zoonotic bacterial agents in rodents and small mammals in Iran
Source: Eur J Public Health. 2025 Jan 13;35(Suppl 1):i41–7. doi: 10.1093/eurpub/ckae132 (PMC11725960; doi:10.1093/eurpub/ckae132)
Supplement: ckae132_Supplementary_Data [file ckae132_supplementary_data.zip › ckae132_Supplementary_Data/ejph-2024-02--0075-File006.docx]

**TableS1. Oligonucleotide sequences Primer and probe used in this study for quantitative real-time PCR (qPCR) analysis.**

| Organism | Gene target | Sequence of Primers and probe (5′- 3′) | Reference |
| --- | --- | --- | --- |
| *Coxiella burnetii* | IS1111 | F: 5′-AAAACGGATAAAAAGAGTCTGTGGTT-3′ | (1) |
|  |  | R: 5′-CCACACAAGCGCGATTCAT-3′ |  |
|  |  | Probe: 5′-6-FAM-AAAGCACTCATTGAGCGCCGCG-TAMRA-3′ |  |
| *Rickettsia* spp. | 16srRNA | F: 5′-CGCAACCCTYATTCTTATTTGC-3′ | (2) |
|  |  | R:5′-CCTCTGTAAACACCATTGTAGCA-3′ |  |
|  |  | Probe: 5′-6- FAM-TAAGAAAACTGCCGGTGATAAGCCGGAG–TAMRA-3′ |  |
| *Bartonella spp.* | 16S-23S rRNA | F: 5′-GGGGAAGGTTTTCCGGTTTATC-3′ | (3) |
|  |  | R:5′-GAGGACTTGAACCTCCGACC-3′ |  |
|  |  | Probe: 5′-6FAM'-GGAGGGCTTGTAGCTCAGYTGGTTAGAGCG_TAMRA-3′ |  |
| *Brucella spp.* | IS711 | F: 5′-GCTTGAAGCTTGCGGACAGT-3′ | (4) |
|  |  | R:5′-GGCCTACCGCTGCGAAT-3′ |  |
|  |  | Probe: 5′-FAM-AAGCCAACACCCGGCCATTATGGT-TAMRA-3′ |  |
| *Ehrlichia* | 16S rRNA | F: 5′-TCGCTATTAGATGAGCCTACGT-3′ | (5) |
|  |  | R:5′-GAGTCTGGACCGTATCTCAGT-3′ |  |
| *Borrelia* | 16S rRNA | F: 5′-GGTCAAGACTGACGCTGAGTCA-3′ | (6) |
|  |  | R:5′-GGCGGCCACTTAACACGTTAG-3′ |  |
|  |  | Probe: 5′-Fam-TCTACGCTGTAAACGATGCACACTTGGTG-BHQ-1-3′ |  |

**Table S2. Primer sequences used for species determination using PCR and multiplex PCR methods in this study**

| Genus | Methods | Gene target | Sequence (5′- 3′) | Amplicon size (bp) | Reference |
| --- | --- | --- | --- | --- | --- |
| *Borrelia* | PCR | *rrs* | Forward: 5′-AGAACTAACGCTGGCAGTG-3′  Reverse: 5′-TGCATAGACTTATATATCCGCC-3′ | 575 | (7) |
| *Ehrlichia* | PCR | 16S rRNA | Forward: 5′ -CTAGAGGTCGAAAGAGGATAG-3′  Reverse: 5′-GTGCTGATTTGACATCATCC-3′ | 555 | (8) |
| *Bartonella* | PCR | *gltA* | Forward: 5′-GCTATGTCTGCATTCTATCA-3′  Reverse: 5′-GATCTTCAATCATTTCTTTCCA-3′ | 790 | (9) |

**References**

1. Schneeberger PM, Hermans MH, van Hannen EJ, Schellekens JJ, Leenders AC, Wever PC. Real-time PCR with serum samples is indispensable for early diagnosis of acute Q fever. Clinical and vaccine immunology. 2010;17(2):286-90.

2. Giulieri S, Jaton K, Cometta A, Trellu LT, Greub G. Development of a duplex real time PCR for the detection of Rickettsia spp. and typhus group rickettsia in clinical samples. FEMS Immunology & Medical Microbiology. 2012;64(1):92-7.

3. Bahari A, Azami S, Goudarztalejerdi A, Karimi S, Esmaeili S, Chomel BB, et al. Focus: Zoonotic Disease: Molecular Detection of Zoonotic Pathogens in the Blood and Tissues of Camels (Camelus dromedarius) in Central Desert of Iran. The Yale Journal of Biology and Medicine. 2021;94(2):249.

4. Hinić V, Brodard I, Thomann A, Cvetnić Ž, Makaya P, Frey J, et al. Novel identification and differentiation of Brucella melitensis, B. abortus, B. suis, B. ovis, B. canis, and B. neotomae suitable for both conventional and real-time PCR systems. Journal of microbiological methods. 2008;75(2):375-8.

5. Peleg O, Baneth G, Eyal O, Inbar J, Harrus S. Use of chimeric DNA-RNA primers in quantitative PCR for detection of Ehrlichia canis and Babesia canis. Applied and environmental microbiology. 2009;75(19):6393-8.

6. Ornstein K, Barbour AG. A reverse transcriptase–polymerase chain reaction assay of Borrelia burgdorferi 16S rRNA for highly sensitive quantification of pathogen load in a vector. Vector-Borne & Zoonotic Diseases. 2006;6(1):103-12.

7. Wodecka B, Leońska A, Skotarczak B. A comparative analysis of molecular markers for the detection and identification of Borrelia spirochaetes in Ixodes ricinus. Journal of medical microbiology. 2010;59(3):309-14.

8. Zhai J, Wu Y, Chen J, Zou J, Shan F, Li W, et al. Identification of Amblyomma javanense and detection of tick-borne Ehrlichia spp. in confiscated Malayan Pangolins. International Journal for Parasitology: Parasites and Wildlife. 2021;14:107-16.

9. Bai Y, Osikowicz LM, Kosoy MY, Eisen RJ, Atiku LA, Mpanga JT, et al. Comparison of zoonotic bacterial agents in fleas collected from small mammals or host-seeking fleas from a Ugandan region where plague is endemic. Msphere. 2017;2(6):10.1128/msphere. 00402-17.

| **TableS3. Geographical occurrence of the studied small mammals in the present study and the infected specimens with *Coxiella burnetii, Bartonella, Rickettsia, Ehrlichia, Brucella,* and *Borrelia*** **in the samples collected between 2016 and 2020 in Iran.** | | | | | | | | | | | |  |
| --- | --- | --- | --- | --- | --- | --- | --- | --- | --- | --- | --- | --- |
| Genus | Species | Province | | | | | | | | | |  |
|  |  | Hormozgan (%) | Kurdistan (%) | Qazvin (%) | Lorestan (%) | Golestan (%) | Guilan (%) | West Azerbaijan (%) | East Azerbaijan (%) | Tehran (%) | Hamedan (%) | Total |
| *Acomys* | *dimidiatus* | 10 (100) | 0 (0) | 0 (0) | 0 (0) | 0 (0) | 0 (0) | 0 (0) | 0 (0) | 0 (0) | 0 (0) | 10 (1.61) |
| *Apodemus* | *uralensis* | 0 (0) | 0 (0) | 0 (0) | 0 (0) | 1 (33.33) | 2 (66.66) | 0 (0) | 0 (0) | 0 (0) | 0 (0) | 45 (7.28) |
|  | *witherbyi* | 0 (0) | 3 (11.11) | 4 (14.81) | 0 (0) | 0 (0) | 0 (0) | 0 (0) | 16 (59.25) | 4 (14.81) | 0 (0) |  |
|  | *ponticus* | 0 (0) | 1 (50.00) | 0 (0) | 1 (50.00) | 0 (0) | 0 (0) | 0 (0) | 0 (0) | 0 (0) | 0 (0) |  |
|  | sp*.* | 0 (0) | 13 (100) | 0 (0) | 0 (0) | 0 (0) | 0 (0) | 0 (0) | 0 (0) | 0 (0) | 0 (0) |  |
| *Arvicola* | *persicus* | 0 (0) | 11 (36.66) | 6 (20.00) | 1 (3.33) | 0 (0) | 0 (0) | 0 (0) | 8 (26.66) | 0 (0) | 4 (13.33) | 30 (4.85) |
| *Chionomys* | *nivalis* | 0 (0) | 0 (0) | 0 (0) | 0 (0) | 0 (0) | 0 (0) | 0 (0) | 2 (100) | 0 (0) | 0 (0) | 2 (0.32) |
| *Nothocricetulus* | *migratorius* | 0 (0) | 1 (12.50) | 2 (25.00) | 1 (12.50) | 0 (0) | 0 (0) | 0 (0) | 3 (37.5) | 0 (0) | 1 (12.50) | 8 (1.29) |
| *Dryomys* | *nitedula* | 0 (0) | 0 (0) | 0 (0) | 0 (0) | 0 (0) | 0 (0) | 0 (0) | 7 (100) | 0 (0) | 0 (0) | 7 (1.13) |
| *Ellobius* | *lutescens* | 0 (0) | 0 (0) | 0 (0) | 0 (0) | 0 (0) | 0 (0) | 0 (0) | 1 (16.66) | 0(0) | 5 (83.33) | 6 (0.97) |
| *Meriones* | *libycus* | 0 (0) | 0 (0) | 0 (0) | 0 (0) | 0 (0) | 0 (0) | 0 (0) | 1 (4.54) | 0 (0) | 21 (95.45) | 315 (50.97) |
|  | *persicus* | 1 (0.40) | 3 (1.21) | 49 (19.91) | 10 (4.06) | 0 (0) | 0 (0) | 0 (0) | 42 (17.07) | 12 (4.87) | 129 (52.43) |  |
|  | *tristrami* | 0 (0) | 1 (5.26) | 0 (0) | 14 (73.68) | 0 (0) | 0 (0) | 0 (0) | 0 (0) | 0 (0) | 4 (21.05) |  |
|  | *vinogradovi* | 0 (0) | 0 (0) | 5 (17.85) | 0 (0) | 0 (0) | 0 (0) | 7 (25.00) | 10 (35.71) | 0 (0) | 6 (21.42) |  |
| *Microtus* | *schidlovskii* | 0 (0) | 11 (100) | 0 (0) | 0 (0) | 0 (0) | 0 (0) | 0 (0) | 0 (0) | 0 (0) | 0 (0) | 101 (16.34) |
|  | *karamani* | 0 (0) | 1 (100) | 0 (0) | 0 (0) | 0 (0) | 0 (0) | 0 (0) | 0 (0) | 0 (0) | 0 (0) |  |
|  | *mystacinus* | 0 (0) | 0 (0) | 0 (0) | 0 (0) | 0 (0) | 0 (0) | 0 (0) | 1 (100) | 0 (0) | 0 (0) |  |
|  | *obscurus* | 0 (0) | 0 (0) | 0 (0) | 0 (0) | 0 (0) | 1 (100) | 0 (0) | 0 (0) | 0 (0) | 0 (0) |  |
|  | *paradoxus* | 0 (0) | 0 (0) | 0 (0) | 0 (0) | 2 (100) | 0 (0) | 0 (0) | 0 (0) | 0 (0) | 0 (0) |  |
|  | *qazvinensis* | 0 (0) | 22 (34.92) | 31 (49.20) | 1 (1.58) | 0 (0) | 0 (0) | 0 (0) | 0 (0) | 0 (0) | 9 (14.28) |  |
|  | *socialis* | 0 (0) | 0 (0) | 0 (0) | 3 (14.28) | 0 (0) | 0 (0) | 0 (0) | 13(61.90) | 5 (23.80) | 0 (0) |  |
|  | cf. *irani* | 0 (0) | 0 (0) | 0 (0) | 1 (100) | 0 (0) | 0 (0) | 0 (0) | 0 (0) | 0 (0) | 0 (0) |  |
| *Rattus* | *rattus* | 23 (92.00) | 0 (0) | 0 (0) | 0 (0) | 0 (0) | 2 (8.00) | 0 (0) | 0 (0) | 0 (0) | 0 (0) | 26 (4.20) |
|  | *norvegicus* | 0 (0) | 0 (0) | 0 (0) | 0 (0) | 0 (0) | 0 (0) | 0 (0) | 1 (100) | 0 (0) | 0 (0) |  |
| *Mustela* | *nivalis* | 0 (0) | 0 (0) | 0 (0) | 0 (0) | 0 (0) | 0 (0) | 0 (0) | 1 (100) | 0 (0) | 0 (0) | 1 (0.16) |
| *Nesokia* | *indica* | 2 (25.00) | 0 (0) | 0 (0) | 0 (0) | 6 (75.00) | 0 (0) | 0 (0) | 0 (0) | 0 (0) | 0 (0) | 8 (1.29) |
| *Tatera* | *indica* | 19 (100) | 0 (0) | 0 (0) | 0 (0) | 0 (0) | 0 (0) | 0 (0) | 0 (0) | 0 (0) | 0 (0) | 19 (3.07) |
| *Scarturus* | *indicus* | 0 (0) | 0 (0) | 1 (100) | 0 (0) | 0 (0) | 0 (0) | 0 (0) | 0 (0) | 0 (0) | 0 (0) | 5 (0.80) |
|  | *williamsi* | 0 (0) | 0 (0) | 2 (50.00) | 0 (0) | 0 (0) | 0 (0) | 0 (0) | 2 (50.00) | 0 (0) | 0 (0) |  |
| *Mus* | *macedonicus* | 0 (0) | 2 (10.00) | 0 (0) | 16 (80.00) | 0 (0) | 0 (0) | 0 (0) | 2 (10.00) | 0 (0) | 0 (0) | 32 (5.17) |
|  | *musculus* | 5 (41.66) | 0 (0) | 1 (8.33) | 0 (0) | 1 (8.33) | 4 (33.33) | 0 (0) | 1 (8.33) | 0 (0) | 0 (0) |  |
| *Crocidura* | *caspica* | 0 (0) | 0 (0) | 0 (0) | 0 (0) | 0 (0) | 1 (100) | 0 (0) | 0 (0) | 0 (0) | 0 (0) | 3 (0.48) |
|  | *suaveolens* | 0 (0) | 0 (0) | 1 (50.00) | 0 (0) | 1 (50.00) | 0 (0) | 0 (0) | 0 (0) | 0 (0) | 0 (0) |  |
| Total | | 60 (9.70) | 69 (11.16) | 102 (16.50) | 48 (7.76) | 11 (1.77) | 10 (1.61) | 7 (1.13) | 111 (17.96) | 21 (3.39) | 179 (28.96) | 618 (100) |

| **Number (%) of positive samples** | | | | | | **No. tested** | **Province** |
| --- | --- | --- | --- | --- | --- | --- | --- |
| *Ehrlichia* spp. (%) | *Rickettsia* spp. (%) | *Borrelia* spp. (%) | *Brucella* spp. (%) | *Coxiella burnetii* (%) | *Bartonella* spp. (%) |  |  |
| 0(0) | 0 (0) | 1 (0.55) | 3 (1.77) | 2 (1.11) | 176 (98.32) | 179 | Hamedan |
| 1 (0.98) | 0 (0) | 0 (0) | 0 (0) | 0 (0) | 99 (97.05) | 102 | Qazvin |
| 3 (4.34) | 0 (0) | 0 (0) | 0 (0) | 0 (0) | 40 (66.66) | 60 | Hormozgan |
| 1(1.44) | 0 (0) | 0 (0) | 0 (0) | 1 (1.66) | 64 (92.75) | 69 | Kurdistan |
| 0 (0) | 0 (0) | 0 (0) | 0 (0) | 0 (0) | 26 (54.16) | 48 | Lorestan |
| 0 (0) | 0 (0) | 0 (0) | 0 (0) | 0 (0) | 17 (80.95) | 21 | Tehran |
| 0 (0) | 0 (0) | 0 (0) | 0 (0) | 0 (0) | 4 (40.00) | 10 | Guilan |
| 8 (7.20) | 0 (0) | 4 (3.60) | 0 (0) | 1 (0.90) | 99 (89.18) | 111 | East Azerbaijan |
| 1 (14.28) | 0 (0) | 0 (0) | 0 (0) | 0 (0) | 7 (100) | 7 | West Azerbaijan |
| 1 (9.09) | 0 (0) | 0 (0) | 0 (0) | 0 (0) | 0 (0) | 11 | Golestan |
| **15 (2.42)** | **0 (0)** | **5 (0.80)** | **3 (0.48)** | **4 (0.64)** | **532(86.08)** | **618** | **Total** |

**Table S4. The molecular investigation of *Coxiella burnetii, Bartonella, Rickettsia, Ehrlichia, Brucella,* and *Borrelia* pathogens in small mammals of different provinces of Iran captured during the period of 2016 to 2020**

| **Table S5- The prevalence of infection caused by *Coxiella burnetii, Bartonella, Rickettsia, Ehrlichia, Brucella,* and *Borrelia* in studied small mammals collected from 2016 to 2020 in various regions of Iran** | | | | | | | | |
| --- | --- | --- | --- | --- | --- | --- | --- | --- |
| **Genus** | **Species** | **No.** | **Number (%) of positive sample** | | | | | |
|  |  |  | *C. burnetii* | *Bartonella* spp. | *Brucella* spp. | *Borrelia* spp. | *Ehrlichia* spp. | *Rickettsia* spp. |
| *Acomys* | *dimidiatus* | 10 | 0 (0) | 1 (10.00) | 0 (0) | 0 (0) | 0 (0) | 0 (0) |
| *Apodemus* | *uralensis* | 3 | 0 (0) | 1 (33.33) | 0 (0) | 0 (0) | 0 (0) | 0 (0) |
|  | *witherbyi* | 27 | 1 (3.70) | 21(77.77) | 0 (0) | 0 (0) | 0 (0) | 0 (0) |
|  | *ponticus* | 2 | 0 (0) | 2 (100) | 0 (0) | 0 (0) | 0 (0) | 0 (0) |
|  | sp. | 13 | 0 (0) | 11 (84.61) | 0 (0) | 0 (0) | 0 (0) | 0 (0) |
| *Arvicola* | *persicu*s | 30 | 1 (3.33) | 27 (90.00) | 0 (0) | 0 (0) | 0 (0) | 0 (0) |
| *Chionomys* | *nivalis* | 2 | 0 (0) | 2 (100) | 0 (0) | 0 (0) | 1 (50.00) | 0 (0) |
| *Nothocricetulus* | *migratorius* | 8 | 0 (0) | 6 (75.00) | 0 (0) | 0 (0) | 0 (0) | 0 (0) |
| *Dryomys* | *nitedula* | 7 | 0 (0) | 4 (57.14) | 0 (0) | 0 (0) | 0 (0) | 0 (0) |
| *Ellobius* | *lutescens* | 6 | 0 (0) | 6 (100) | 0 (0) | 0 (0) | 0 (0) | 0 (0) |
| *Meriones* | *libycus* | 22 | 0 (0) | 22 (100) | 0 (0) | 0 (0) | 0 (0) | 0 (0) |
|  | *persicus* | 246 | 2 (0.81) | 235 (95.52) | 2 (0.81) | 5 (2.03) | 9 (3.65) | 0 (0) |
|  | *tristrami* | 19 | 0 (0) | 13 (68.42) | 0 (0) | 0 (0) | 0 (0) | 0 (0) |
|  | *vinogradovi* | 28 | 0 (0) | 27 (96.42) | 0 (0) | 0 (0) | 0 (0) | 0 (0) |
| *Microtus* | *schidlovskii* | 11 | 0 (0) | 11 (100) | 0 (0) | 0 (0) | 0 (0) | 0 (0) |
|  | *karamani* | 1 | 0 (0) | 1 (100) | 0 (0) | 0 (0) | 0 (0) | 0 (0) |
|  | *mystacinus* | 1 | 0 (0) | 1 (100) | 0 (0) | 0 (0) | 0 (0) | 0 (0) |
|  | *obscurus* | 1 | 0 (0) | 0 (0) | 0 (0) | 0 (0) | 0 (0) | 0 (0) |
|  | *paradoxus* | 2 | 0 (0) | 0 (0) | 0 (0) | 0 (0) | 0 (0) | 0 (0) |
|  | *qazvinensis* | 63 | 0 (0) | 59 (93.65) | 1 (1.58) | 0 (0) | 1 (1.58) | 0 (0) |
|  | *socialis* | 21 | 0 (0) | 18 (85.71) | 0 (0) | 0 (0) | 0 (0) | 0 (0) |
|  | cf. *irani* | 1 | 0 (0) | 0 (0) | 0 (0) | 0 (0) | 0 (0) | 0 (0) |
| *Rattus* | *rattus* | 25 | 0 (0) | 16 (64.00) | 0 (0) | 0 (0) | 1 (4.00) | 0 (0) |
|  | *norvegicus* | 1 | 0 (0) | 1 (100) | 0 (0) | 0 (0) | 0 (0) | 0 (0) |
| *Mustela* | *nivalis* | 1 | 0 (0) | 0 (0) | 0 (0) | 0 (0) | 0 (0) | 0 (0) |
| *Nesokia* | *indica* | 8 | 0 (0) | 2 (25.00) | 0 (0) | 0 (0) | 0 (0) | 0 (0) |
| *Tatera* | *indica* | 19 | 0 (0) | 18 (94.73) | 0 (0) | 0 (0) | 1 (5.26) | 0 (0) |
| *Scarturus* | *indicus* | 1 | 0 (0) | 1 (100) | 0 (0) | 0 (0) | 0 (0) | 0 (0) |
|  | *williamsi* | 4 | 0 (0) | 3 (75.00) | 0 (0) | 0 (0) | 0 (0) | 0 (0) |
| *Mus* | *macedonicus* | 20 | 0 (0) | 17 (85.00) | 0 (0) | 0 (0) | 0 (0) | 0 (0) |
|  | *musculus* | 12 | 0 (0) | 5 (41.66) | 0 (0) | 0 (0) | 2 (16.66) | 0 (0) |
| *Crocidura* | *caspica* | 1 | 0 (0) | 1 (100) | 0 (0) | 0 (0) | 0 (0) | 0 (0) |
|  | *suaveolens* | 2 | 0 (0) | 0 (0) | 0 (0) | 0 (0) | 0 (0) | 0 (0) |
| **Total** | | **618** | **4 (0.64)** | **532 (86.08)** | **3 (0.48)** | **5 (0.80)** | **15 (2.42)** | **0 (0)** |
